# A Virtual Pediatrics Elective for Underrepresented in Medicine Medical Students: An Innovative Residency Recruitment Tool to Promote Equity and Community Building

**DOI:** 10.7759/cureus.104984

**Published:** 2026-03-10

**Authors:** Zarina S Norton, Valeria C Cohran, Jennifer L Trainor

**Affiliations:** 1 Pediatrics, University of Michigan, Ann Arbor, USA; 2 Pediatrics, Northwestern University Feinberg School of Medicine, Chicago, USA

**Keywords:** inclusion in residency, pediatric residency recruitment, underrepresented in medicine, virtual elective, virtual rotation

## Abstract

Background

Residency programs may use in-person medical student visiting rotations to highlight aspects of their programs and recruit diverse residents. Medical students, especially those underrepresented in medicine (URIM), may use visiting rotations to understand program culture, network, and to demonstrate their skills to residency programs. However, financial costs and time constraints during medical school training may limit some students’ ability to participate in in-person visiting rotations.

Objective

We developed an innovative, fully virtual rotation for URIM students to provide pediatric education, mentorship, and introduction/immersion into our residency program, with the goals of providing a valuable educational experience and increasing recruitment of diverse residents.

Methods

We implemented a two-week virtual pediatrics rotation at the Ann & Robert H. Lurie Children’s Hospital of Chicago, incorporating two to four hours per day of virtual interaction, including simulation, educational conferences, mentorship, and meetings with residency/institutional leadership and committees. The elective was offered from 2020 to 2023. Participating students completed post-rotation surveys, rating each session (Likert scale, 1 = poor to 5 = excellent) to evaluate satisfaction with the rotation. Median scores were calculated. Secondary outcomes included the number of elective participants who matched into our pediatrics residency program and the annual proportion of URIM residents matched into our program. In addition, participants who ultimately matched into our residency program completed a long-term post-elective survey one to three years after the elective (sent in 2024), assessing the elective's influence on residency selection, sense of belonging, and mentorship during residency.

Results

From 2020 to 2023, 13 students enrolled in the virtual rotation, and all 13 completed post-rotation surveys. Students rated 23 of 25 sessions (93%) during the elective as “excellent” (median Likert score 5), and all 13 respondents (100%) rated the overall course as “excellent” (Likert score 5). Nine of 13 students (69%) matched into our residency program. By 2023, 39% of pediatrics interns matched were URIM, representing a greater than 300% increase over four years. Eight of the nine residents (89%) who had participated in the virtual elective completed the long-term post-elective survey. All eight respondents (100%) indicated that the virtual elective positively impacted their decision to match into our residency program and that they would recommend the virtual elective to other URIM students. Only one of eight (12.5%) responded that they had been “extremely likely” to rank our residency program highly prior to the elective, compared to all eight (100%) after completing the elective. Six of eight respondents (75%) felt participating in the virtual elective increased their sense of belonging when starting residency, and six of eight (75%) felt the elective made it easier for them to find mentorship during residency.

Conclusion

An innovative virtual immersion of URIM students into a residency program through a two-week virtual elective was associated with increasing URIM match results and yielded high value to students both during the elective and as they entered residency. Virtual electives may help engage students unable to complete visiting rotations and promote equity, aid in recruitment, and improve the sense of belonging in residents.

## Introduction

The COVID-19 pandemic led to significant challenges in medical education as students were removed from clinical rotations to curb viral spread [1,2]. In 2020, United States regulatory bodies strongly discouraged visiting student rotations and established a virtual 2020-2021 residency recruitment season [3]. Medical students often use visiting rotations and in-person interviews to evaluate residency program culture, and students underrepresented in medicine (URIM) may place a higher value on program culture, mentorship, and inclusion practices than others when choosing programs [4,5,6,7]. Constraints on in-person opportunities added to existing challenges for students trying to assess these aspects of programs [8,9]. As many residency programs sought to increase resident diversity [9,10,11], visiting student rotation restrictions and virtual interviews limited the ability of residency programs to showcase their programs and culture to prospective URIM students and increased recruitment challenges. 

In response to the COVID-19 pandemic, some programs implemented virtual rotations to fill educational gaps created by the lack of in-person visiting rotations [2,12,13,14]. Few studies have described experiences with virtual rotations for underrepresented in medicine medical students or their impact on residency program recruitment [15,16]. We implemented an innovative, fully virtual elective pediatric rotation for URIM students, hypothesizing that we could deliver a positive educational experience and impact favorably on residency program recruitment of diverse residents.

## Materials and methods

Rotation development

Based on input from residency program leadership and URIM residents, faculty and students, we developed the two-week elective rotation with the following goals: 1) provide education on core pediatric topics in preparation for residency, 2) connect students with resident and faculty mentors with similar backgrounds and/or interests, 3) create a forum to discuss URIM-centered diversity and health equity topics, and 4) virtually immerse students into our residency program to introduce them to the breadth of clinical, educational, research, advocacy and health equity opportunities and resources.

This study was conducted at Ann & Robert H. Lurie Children’s Hospital of Chicago (Northwestern University, Chicago, IL). The Northwestern University Feinberg School of Medicine Curriculum Committee approved the rotation in August 2020. We offered two separate two-week, for-credit rotations in September 2020 and October 2020, with each block open to up to 10 fourth-year URIM students at accredited medical schools participating in the AAMC Visiting Student Learning Opportunities (VSLO). We capped the number of openings per block to 10 students to better allow for interactivity and participation, given the virtual platform. Based on continued enthusiasm for the virtual elective even after in-person visiting rotations were reinstituted, we offered the two-week elective to fourth-year URIM medical students again once in September 2021 and once in October 2022. For this elective, our medical school defined the following groups as URIM: Black (African/African American), Hispanic/Latino, Native American, Native Hawaiian, Pacific Islander, LGBTQ+, and from economically disadvantaged backgrounds (self-reported). We advertised the rotation via the Council for Medical Student Education in Pediatrics (COMSEP) and Association of Pediatric Program Directors (APPD) listservs, through social media, and by word of mouth.

**Figure 1:**
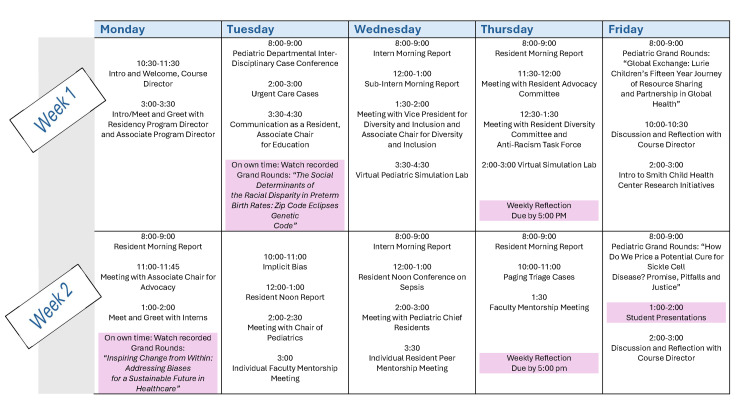
Sample rotation schedule

We created a rotation structure and content using principles of multimedia learning as a conceptual framework [17]. The schedule included two to four hours of virtual interaction daily with approximately two hours of independent study per week, weekly written reflections, and end-of-rotation presentations on a topic of the student’s choice (Figure 1). We connected each student with faculty and resident mentors based on interests. A detailed breakdown of session types, educational formats, and the number of faculty and residents required for each session is included in the Appendix.

A faculty course director and coordinator led the rotation. An additional 20-25 faculty and residents (both URIM and non-URIM) spent one to two hours with students. Students required an internet connection and access to Zoom.

Short-term post-rotation surveys

At the end of each rotation, students completed an anonymous, voluntary online survey. They rated each of the 25 sessions offered during the rotation, as well as the overall course, on a five-point Likert scale (1 = poor, 5 = excellent) and provided optional open-ended comments. We calculated the median rating for each of the 25 sessions and the overall course. We developed the survey with the primary goal of collecting feedback on the rotation and, therefore, did not collect survey validity evidence. We reviewed open-ended responses for feedback but did not perform a formal qualitative analysis.

Residency match results

A secondary outcome was the number of participants in the virtual elective who chose to match into our residency program. We also analyzed overall URIM match results in our residency program, evaluated as the number of matched URIM residents and the percentage of matched pediatric interns identifying as URIM compared to prior years.

Long-term post-rotation surveys

In June 2024, a long-term post-rotation survey was sent to all participants of the virtual elective who ultimately matched into our residency program. The long-term post-rotation survey explored how the virtual elective impacted the residents’ decisions to rank our program highly during the residency match process and how it impacted their ability to find community and mentorship once they started residency.

Statistical analysis

An a priori power analysis was not conducted because the sample size was determined by the number of students enrolled in and completing the curriculum during the study period. All eligible students were invited to participate, representing a convenience sample of the available learner cohort.

This study employed a single-cohort, post-curriculum survey design intended to descriptively evaluate learner perceptions and self-reported outcomes. In the absence of a comparison group, baseline measurement, or experimental manipulation, analyses were primarily descriptive. Results are reported using frequencies, medians, and percentages.

The study was reviewed by the Ann & Robert H. Lurie Children’s Hospital of Chicago Institutional Review Board and was deemed exempt (IRB 2021-4811).

## Results

Student interest and enrollment in rotation

We enrolled 10 URIM students in the virtual elective in 2020 (four in September; six in October). Because of continued interest, we enrolled one student in 2021 and two students in 2022, despite the reinstatement of in-person visiting rotations. All students who applied for the elective through VSLO were enrolled and represented 13 medical schools in 10 states. Fifty-four percent (54%) (seven of 13) identified as Black, 15% (two of 13) as Hispanic/Latino, 15% (two of 13) as mixed race (including Black or Hispanic/Latino), 8% (one of 13) as Pacific Islander, and 8% (one of 13) as coming from an economically disadvantaged background.

Short-term post-rotation surveys

All students who participated in the rotation (n = 13) completed an anonymous post-rotation survey shortly after the rotation (100% response rate). Median scores for 23/25 sessions (93%) were 5 (5 = excellent). One hundred percent (100%) of students rated the overall course 5 (5=excellent). Open-ended comments from short-term post-rotation surveys and weekly reflections were positive, indicating rotation goals were met and providing valuable feedback (Table 1).

**Table 1:** Student comments from anonymous rotation evaluation surveys and course reflections

Topic/session type	Representative student comments
Core Pediatric Topics and Residency Preparation	I really enjoyed the session we had with Dr. X about ‘Communication as a Resident’…She provided practical tips that I will implement in my upcoming rotations and as an intern.
Core Pediatric Topics and Residency Preparation	It was great to learn about general pediatric topics from experts and to learn about complicated cases at daily resident conferences.
Core Pediatric Topics and Residency Preparation	The virtual simulation labs were unique and a great way to learn.
Mentorship	Even over the phone, Dr. X gave me a sense of support and excitement for my future career in pediatrics. He conveyed how much investigational research is being conducted at Lurie Children’s and how there is still so much room to grow.
Mentorship	My first mentorship meeting with Dr. X was so neat … felt very supported.
Introduction to Residency Program	Everyone, faculty and residents, is so consistent and committed to not only a strong learning environment but also a supportive culture.
Introduction to Residency Program	Through dedicated teaching sessions, small group dialogue, resident case reports, and meetings with members of leadership, Lurie has gone above and beyond to show us the personality of the pediatric residency program…. Our virtual experience has clearly demonstrated that the administration is supportive, the trainees are hardworking and happy, and the clinical care provided is thoughtful, evidence-based, and truly high quality for all patients.
Introduction to Residency Program	I was able to learn more about the directions Lurie is planning to take in the future, to address advocacy and community involvement.
Introduction to Residency Program	It has been an overwhelmingly positive experience for me to virtually participate in the Lurie URM elective so far! I am gaining a strong sense of what the residency program values.
Introduction to Residency Program	Everything about my experience on this virtual rotation thus far has been even better than I had hoped. Before it began, I wasn’t sure how much I would be able to ascertain about the people and culture at Lurie via Zoom; however, I really do believe that I’ve been able to get a good sense of what it would be like to be a resident here.
URIM-Centered Equity and Inclusion Topics	I appreciated the honesty and transparency, which the residents shared regarding the efforts to improve recruitment and retention of a diverse workforce, even beyond the residency staff.
URIM-Centered Equity and Inclusion Topics	While it can be overwhelming to know where to start in our effort against racism, I think understanding and addressing our own implicit biases regarding race can be a good first step. I appreciated that Dr. X and Dr. X provided a place to talk about it and gave us practical tools to address it as well.
URIM-Centered Equity and Inclusion Topics	It was eye-opening yet sad to learn about the detrimental effects of racial disparities on birth outcomes.
URIM-Centered Equity and Inclusion Topics	Galvanizing talks like Dr. X’s never fail to re-invigorate my commitment to addressing social determinants of health, pushing for greater health equity, and continuing lifelong learning in cultural humility.
URIM-Centered Equity and Inclusion Topics	I’ve also really appreciated that the administration does not shy away from conversations about diversity and inclusion. When faced with tough questions about representation amongst residents and faculty at the hospital, everyone… handled their responses well, acknowledging the problem at hand, highlighting the importance of building a team of physicians that mirrors the communities they serve, and outlining the institution’s plan to create change.
Collaboration	Truly one of my favorite parts of this elective, though, has been meeting some awesome students from around the country and hearing their unique perspectives as well!
Collaboration	One of my favorite things about the past week on this elective has been the opportunity to think and work as a group.
Collaboration	I hope that the students that I’ve met here will continue to keep in touch for the duration of the application cycle and see each other as a supportive resource as we take the next step towards becoming pediatricians.
Collaboration	This is really the first time as a medical student that I’ve been able to consistently collaborate with trainees at my level from different institutions in a longitudinal and meaningful way. It was particularly meaningful to do this with a group of minority women who, like myself, don’t often feel adequately represented by the medical profession.

Residency URIM match results

We interviewed and subsequently ranked all 13 students who participated in the virtual elective. Participation in the elective did not guarantee the student would match in our program (all participating students' applications were evaluated just as any other applicant to our program). In total, 9 of 13 (81%) of the students completing the virtual rotation from 2020-2023 matched into our pediatric residency program. In 2020, prior to initiating the virtual elective, three of 34 matched pediatrics interns were URIM (9% URIM). In 2021, six of 10 students who completed the virtual elective in 2020 matched with our program, with a total of seven URIM residents out of 34 pediatrics residents (21% URIM) in 2021. In 2022, the one student who completed the virtual elective in 2021 matched with our program, with a total of 10 URIM residents out of 34 pediatrics residents (29% URIM) in 2022. In 2023, both of the two students who completed the virtual elective in 2022 matched with our program, with a total of 14 URIM residents out of 36 pediatrics residents (39% URIM) in 2023. This represents a more than 300% increase in URIM residents matched over four years (Table 2).

**Table 2:** Residency program URIM match results URIM: underrepresented in medicine

Recruitment year	Intern class size	URIM interns	% URIM interns
2019-2020	34	3	9%
2020-2021	34	7	21%
2021-2022	34	10	29%
2022-2023	36	14	39%

Long-term post-rotation surveys

The long-term post-rotation survey was sent to all nine virtual elective participants who matched into our residency program from 2021 to 2023. The survey was sent via Google Forms (Google LLC, Mountain View, CA, USA) in June 2024. Eight of nine (89%) residents who had participated in the virtual elective completed the long-term post-elective survey. At the time, six survey recipients were post-graduate year (PGY)-3 residents, one recipient was a PGY-2 resident, and two survey recipients were PGY-1 residents. Eight of eight respondents (100%) indicated the virtual elective positively impacted their decision to match into our residency program, and 100% indicated they would recommend the virtual elective to other URIM students. Only one of eight (12.5%) responded that they had been “extremely likely” to rank our residency program highly prior to completing the elective, but 8 of 8 (100%) of respondents indicated they were “extremely likely” after completing the elective. Six of eight (75%) respondents felt participating in the virtual elective increased their sense of belonging when starting residency, and six of eight (75%) felt the virtual elective made it easier for them to find mentorship during residency. 

## Discussion

The implementation of a URIM virtual rotation was of high value to participating URIM students and was associated with increasing URIM match results for the residency program. Short-term post-rotation survey results indicated a high level of satisfaction with individual sessions offered during the rotation as well as the whole rotation. When reflecting, all matched residents who completed the long-term post-rotation survey reported an increased likelihood of ranking the residency program highly following completion of the elective. They all indicated they would recommend the rotation to other URIM students, even though in-person rotations had been reinstituted.

Prior studies examining virtual rotations in a variety of medical specialties have similarly demonstrated high learner satisfaction, meaningful interactions with faculty and residents, and perceived value in program exposure, findings that align with our results [12,14,15,16]. Studies have also demonstrated, like ours, that students participating in virtual electives agreed that virtual rotations should be continued, even outside of the COVID-19 pandemic [14].

**Figure 2:**
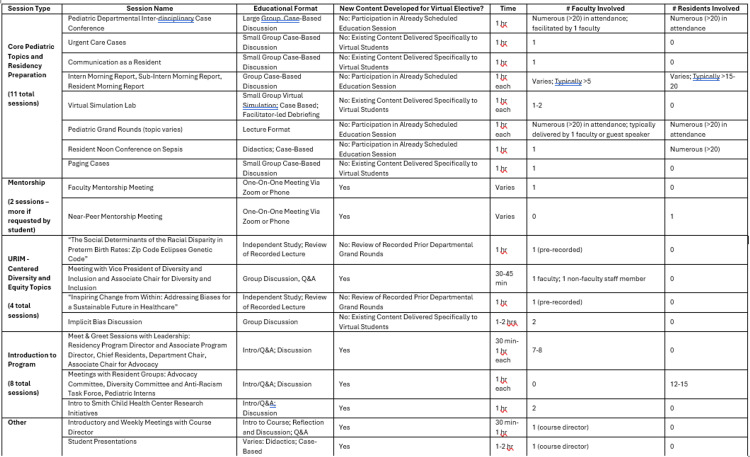
Implementation details for virtual elective by session

A positive unanticipated outcome of our virtual rotation was community building amongst the participating students. As evidenced by student comments immediately after the rotation (Figure 2), they valued the unique opportunity to collaborate and learn with other URIM residents from all over the country. As peer and near-peer mentorship of URIM students has been shown to help address the challenges of underrepresentation in medicine [6,18], this is an important finding.

Additionally, most of the participating students felt the rotation helped increase their sense of belonging and their ability to find mentorship upon starting residency. Efforts to improve URIM recruitment through structured programming, mentorship, and intentional community-building have been shown to positively influence applicants’ perceptions of institutional culture and inclusivity [5,7,9,10,19]. Our findings extend this literature by suggesting that a virtual elective format may serve as an effective platform for delivering these interventions while minimizing financial and logistical barriers. While prior studies on virtual electives have largely focused on short-term satisfaction, our study uniquely explores both immediate and longer-term impacts on recruitment, belonging, mentorship, and community formation among URIM participants.

Limitations included a small sample size at a single program without a direct comparison group. Although residency match results were favorable, we cannot attribute them solely to the virtual rotation, as it was one of several efforts to promote a more inclusive residency program. The increased intern class diversity may reflect a combination of multiple efforts occurring simultaneously. In addition, there may be selection bias, as the students who chose to participate in the elective may have been likely to match into our program for various reasons. However, our results strongly indicate that the virtual elective played a role in our ability to recruit the six URIM virtual elective students who matched into our program in 2021. We believe this established a strong community of URIM residents in our program for the first time, which may have served as a catalyst for future recruitment success. Finally, a formal qualitative analysis of open-ended comments was not performed; however, review of comments provided important feedback for us and for other institutions hoping to implement similar rotations.

## Conclusions

This study suggests that a virtual elective designed for URIM students may be a feasible and meaningful approach to promoting equity in residency recruitment. Virtual rotations offer a low-barrier opportunity for programs to showcase their culture, values, and community while fostering connection, mentorship, and collaboration among participants from diverse institutions.

As financial and logistical barriers can limit access to in-person visiting rotations, virtual electives may help expand opportunities for students who might otherwise be excluded. By creating spaces for community-building and mentorship, virtual rotations have the potential to support URIM students both during the elective experience and beyond, and may serve as a scalable strategy to enhance diversity and inclusion efforts within graduate medical education.
